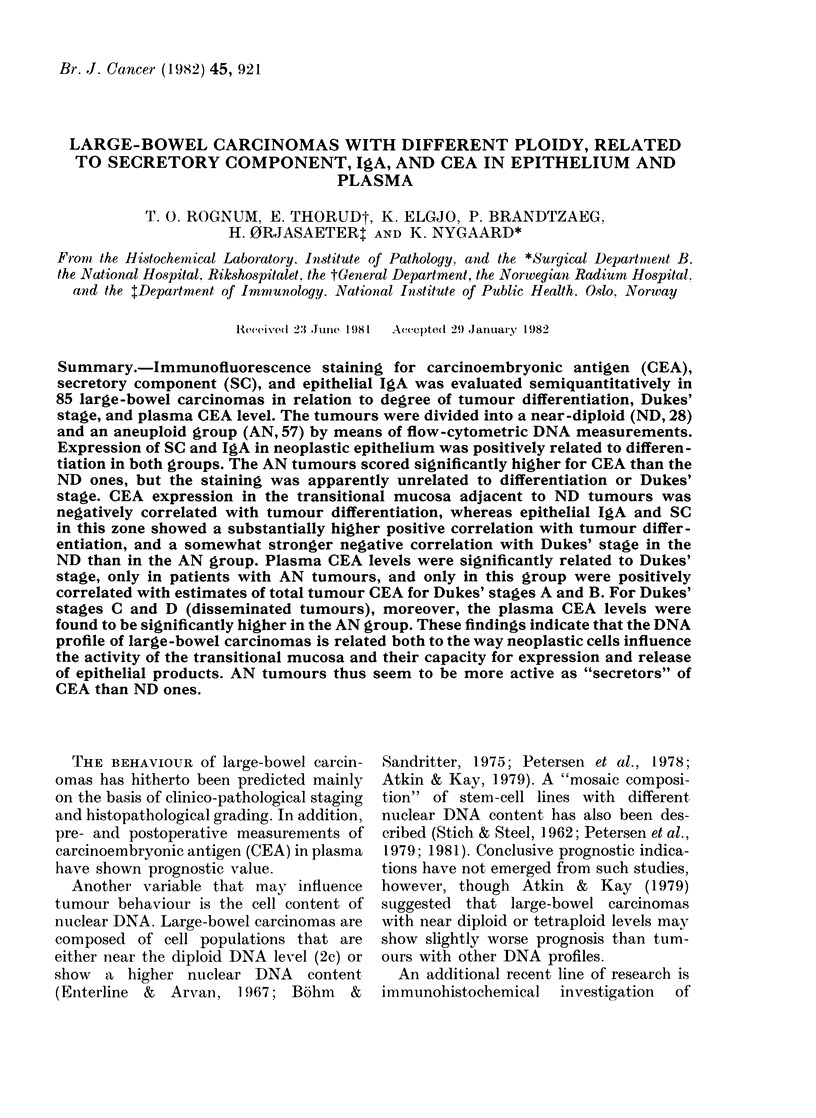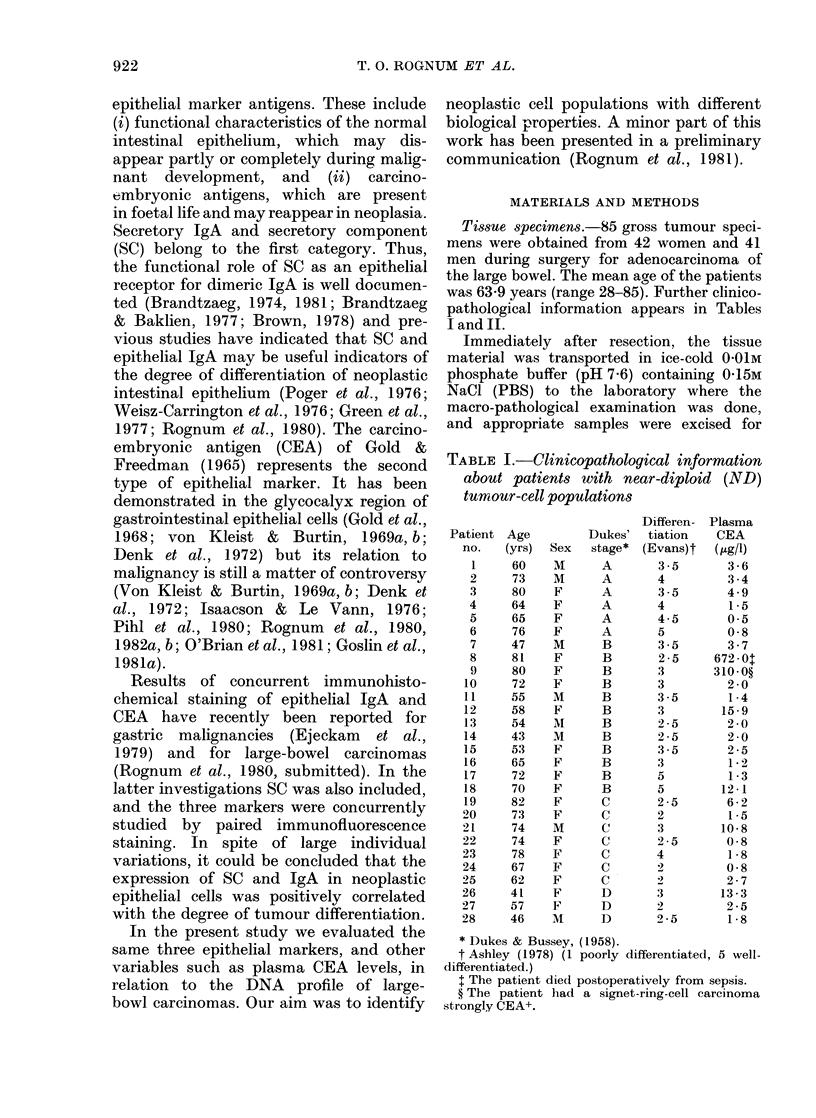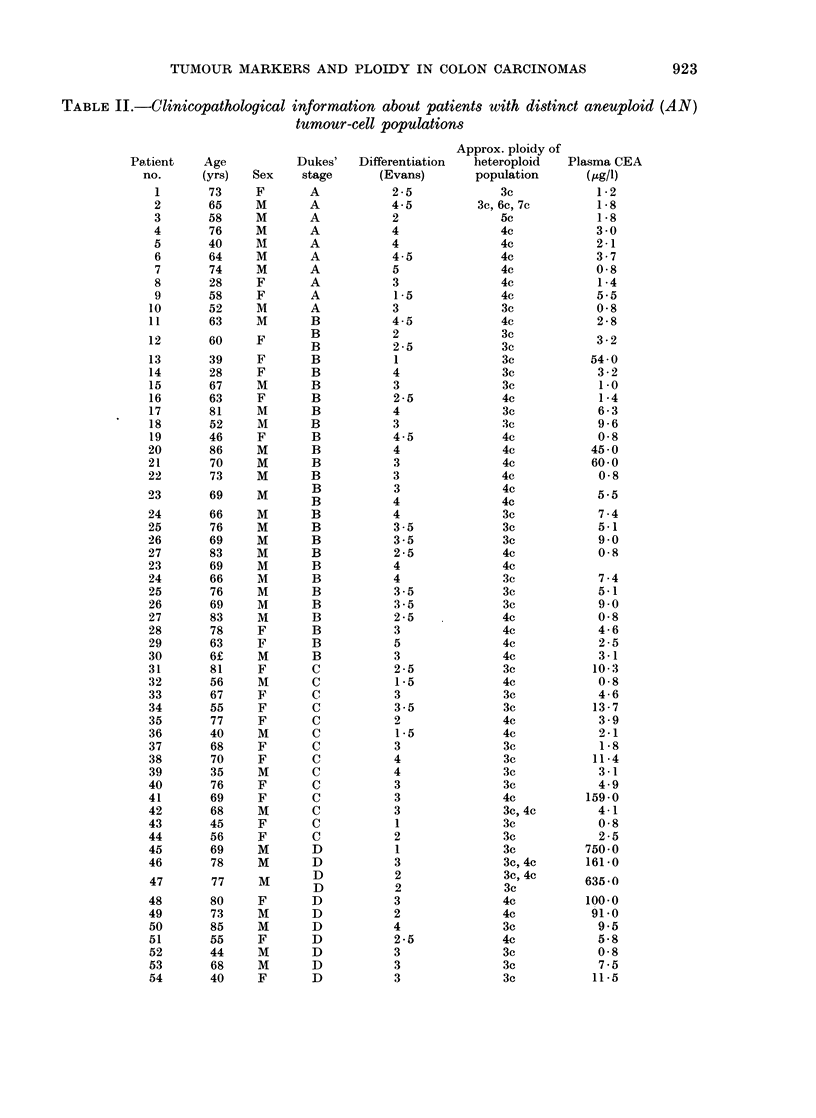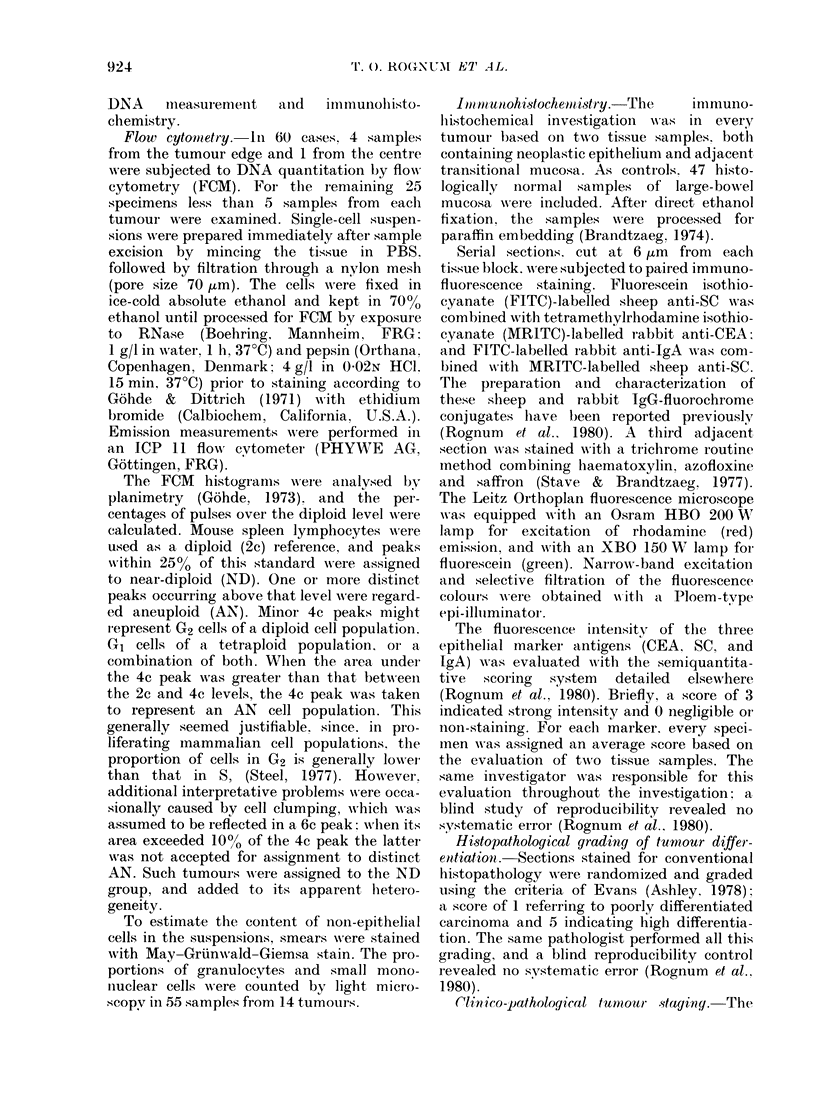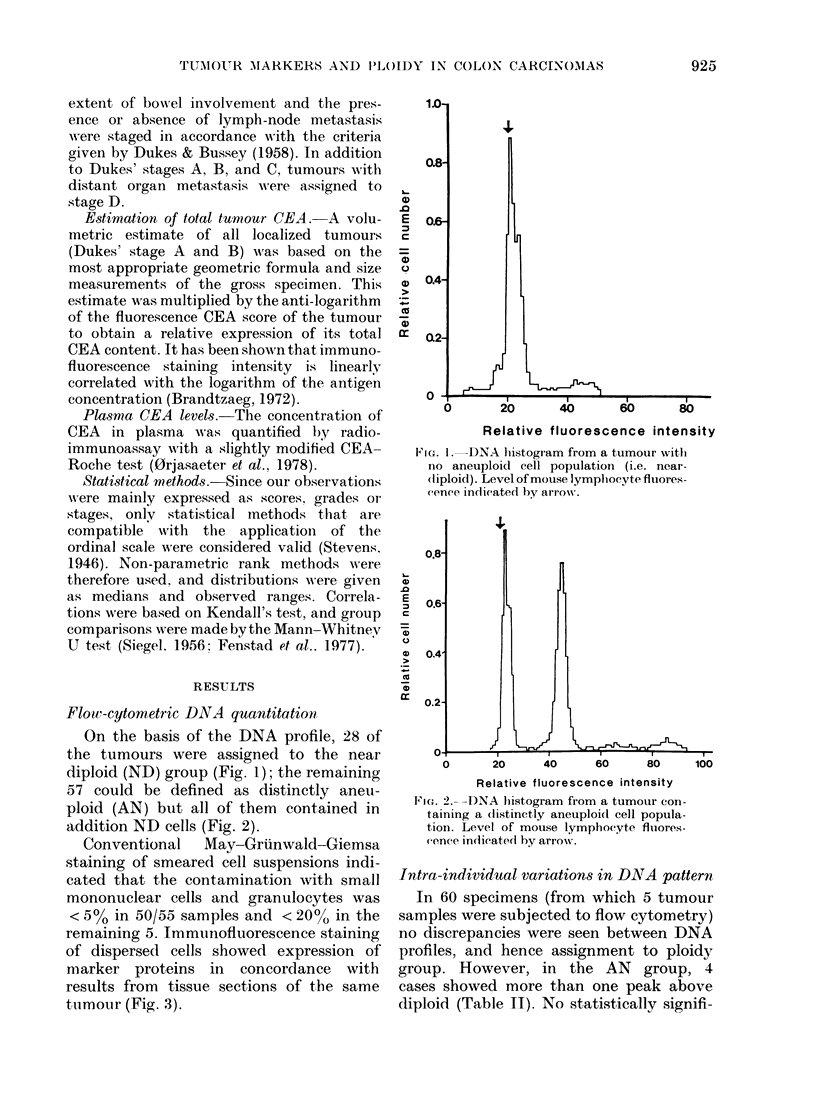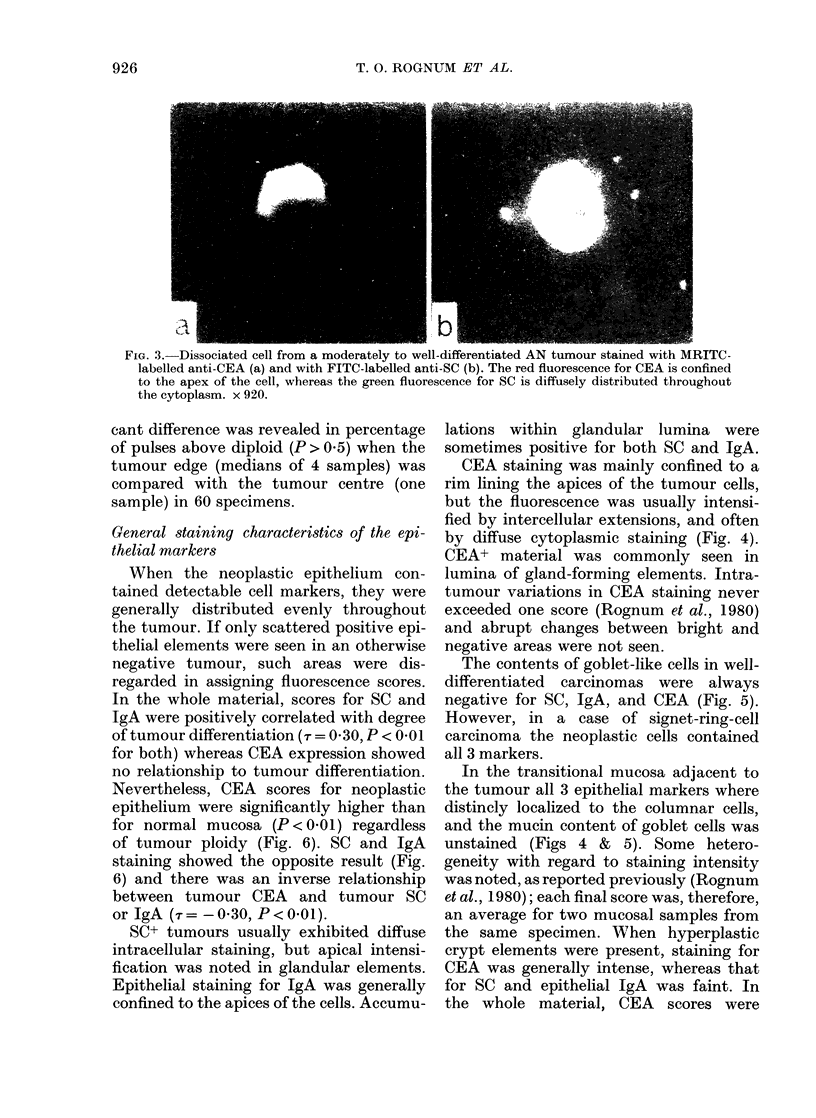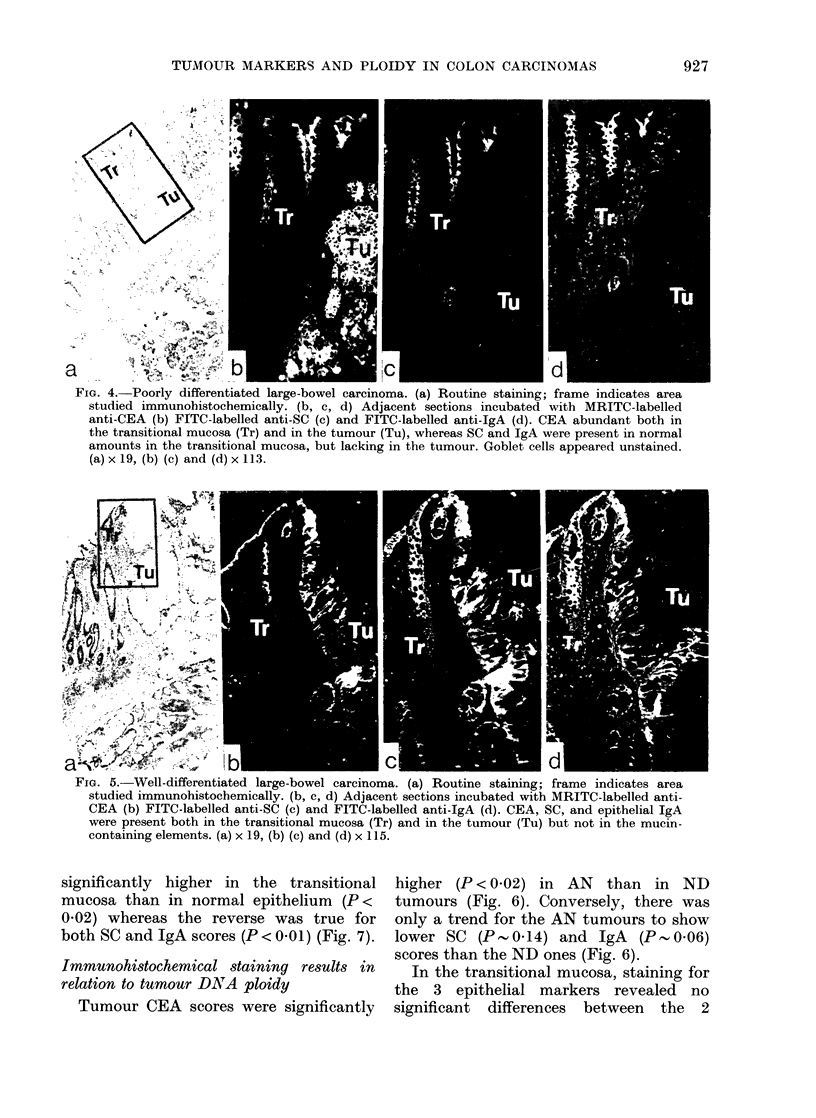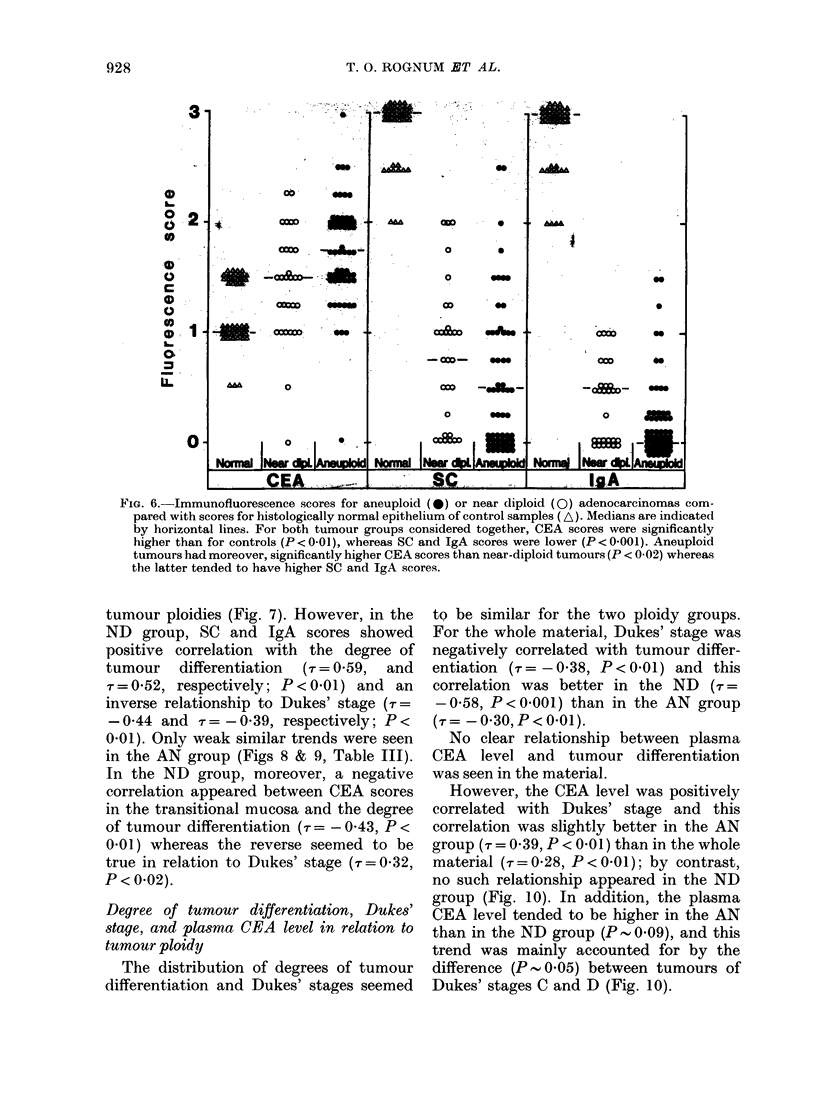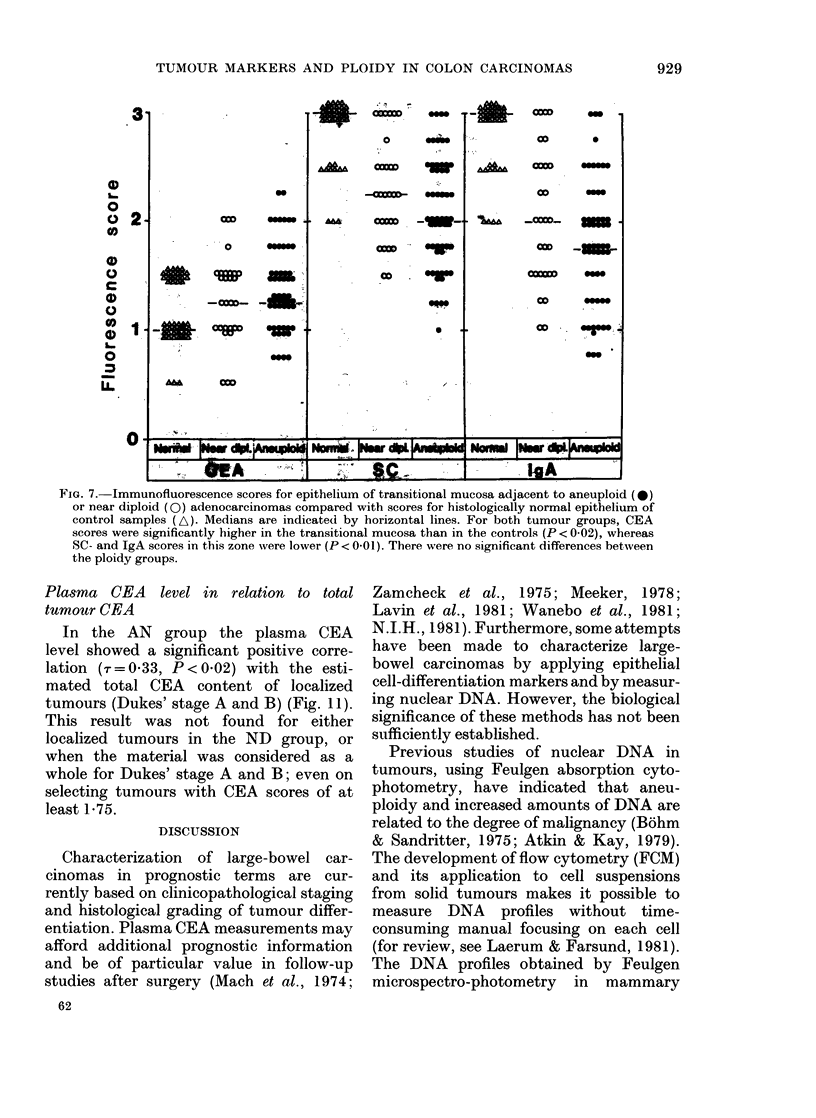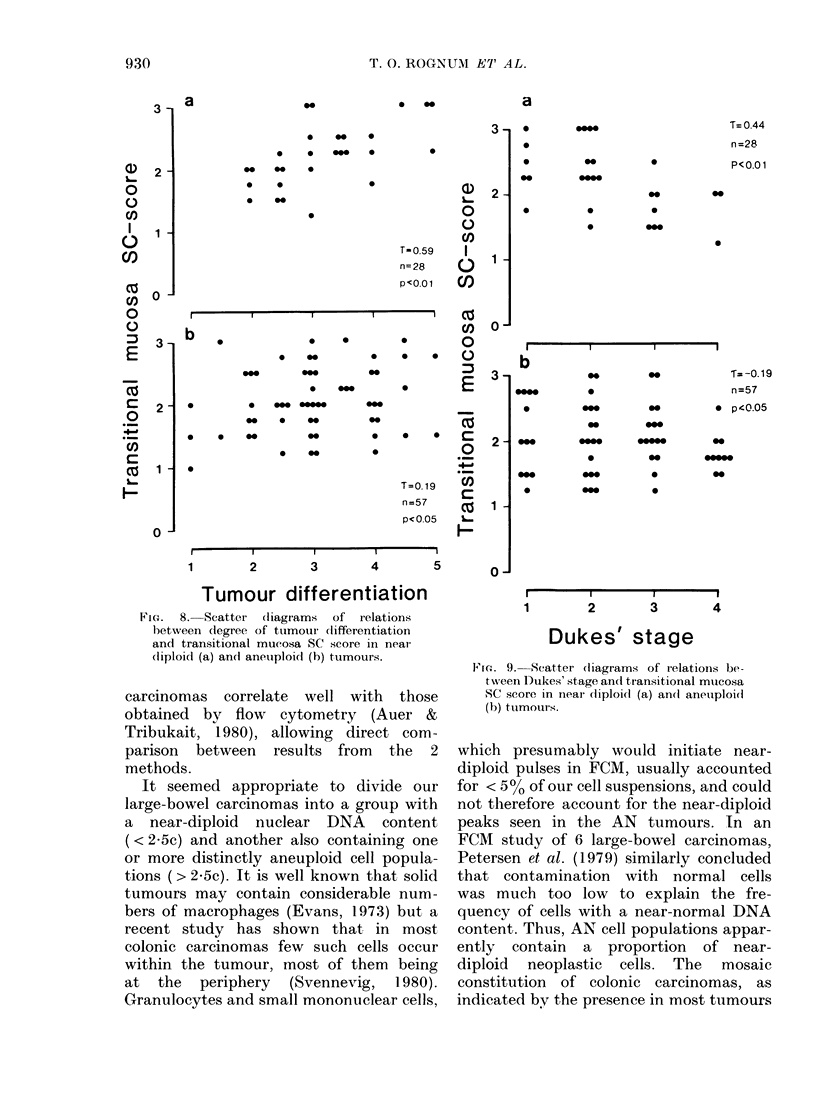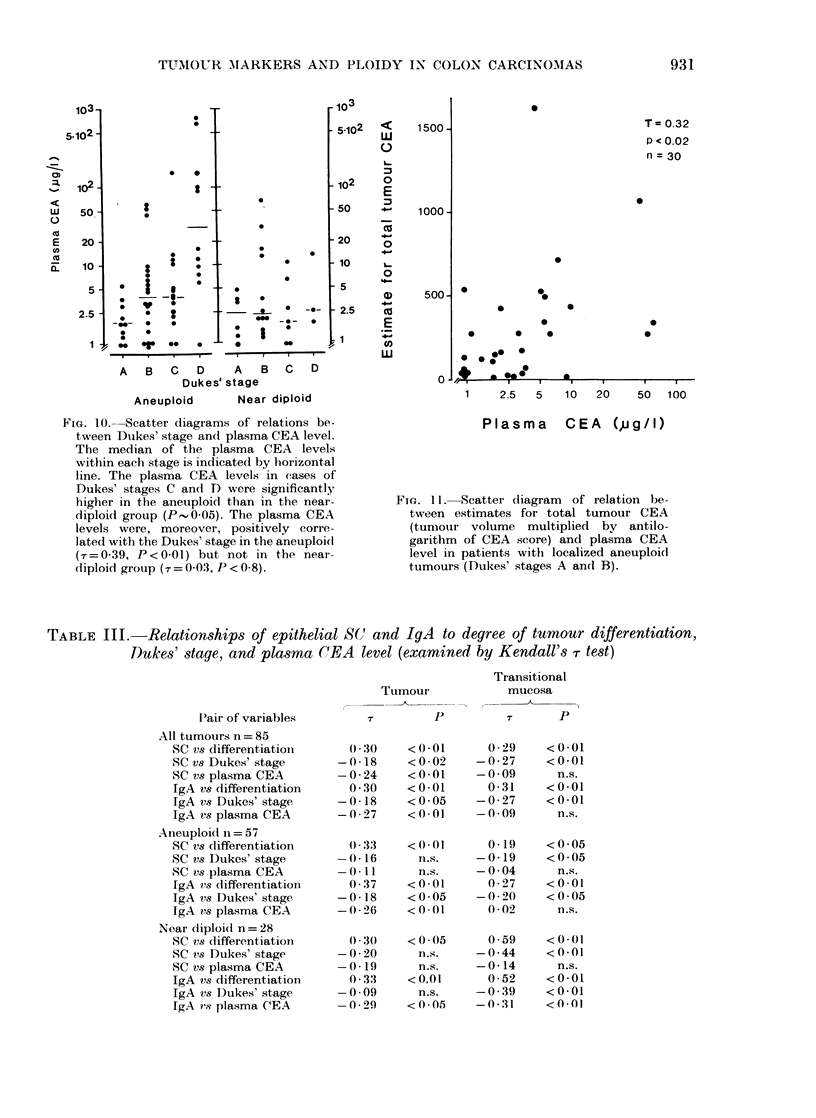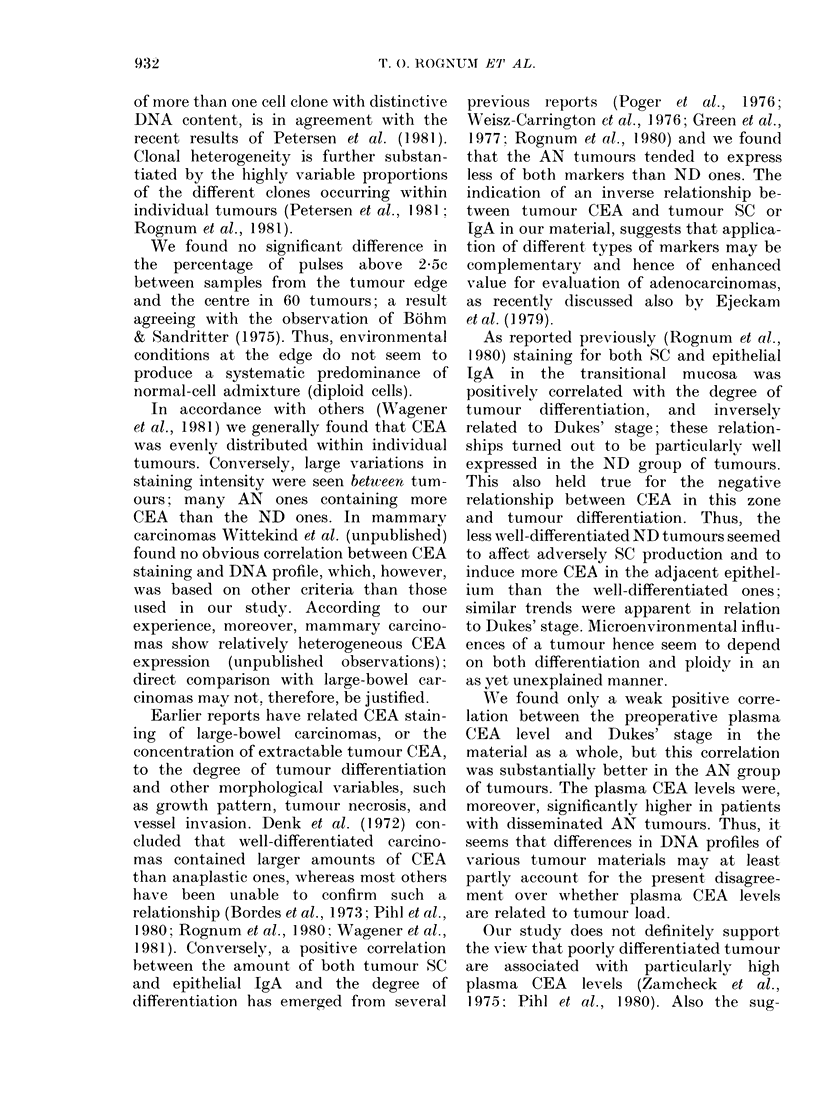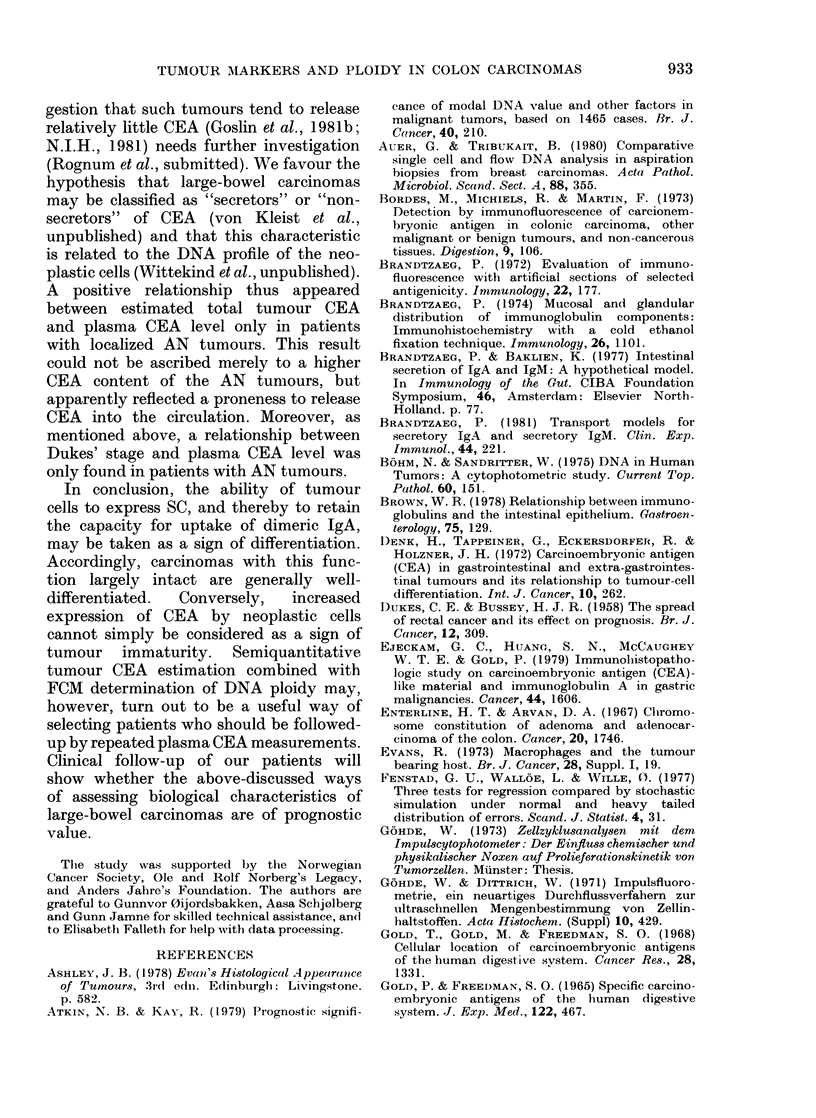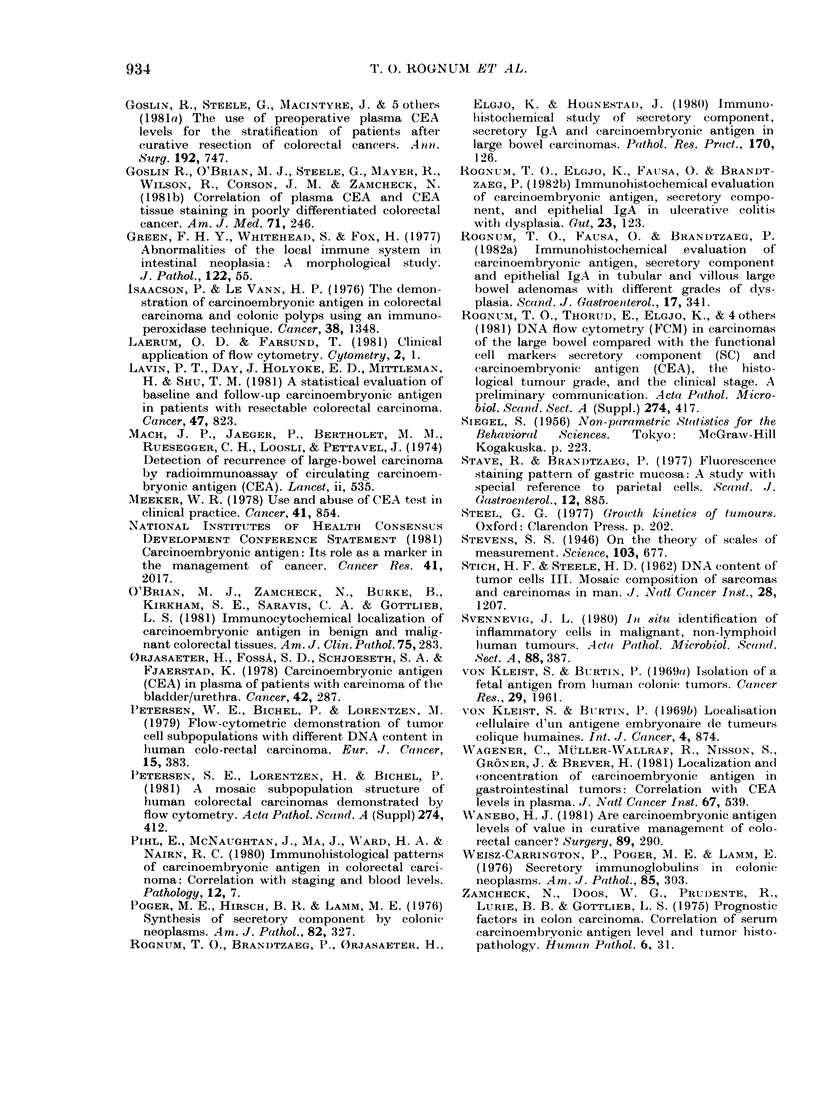# Large-bowel carcinomas with different ploidy, related to secretory component, IgA, and CEA in epithelium and plasma.

**DOI:** 10.1038/bjc.1982.145

**Published:** 1982-06

**Authors:** T. O. Rognum, E. Thorud, K. Elgjo, P. Brandtzaeg, H. Orjasaeter, K. Nygaard

## Abstract

**Images:**


*Br. J. Cancer* (1982) **45**, 921

### LARGE-BOWEL CARCINOMAS WITH DIFFERENT PLOIDY, RELATED TO SECRETORY COMPONENT, IgA, AND CEA IN EPITHELIUM AND PLASMA

T. O. ROGNUM, E. THORUD†, K. ELGJO, P. BRANDTZAEG, H. ØRJASAETER‡ AND K. NYGAARD*

*From the Histochemical Laboratory, Institute of Pathology, and the *Surgical Department B, the National Hospital, Rikshospitalet, the †General Department, the Norwegian Radium Hospital, and the ‡Department of Immunology, National Institute of Public Health, Oslo, Norway*

Received 23 June 1981 Accepted 29 January 1982

**Summary.—Immunofluorescence staining for carcinoembryonic antigen (CEA), secretory component (SC), and epithelial IgA was evaluated semiquantitatively in 85 large-bowel carcinomas in relation to degree of tumour differentiation, Dukes' stage, and plasma CEA level. The tumours were divided into a near-diploid (ND, 28) and an aneuploid group (AN, 57) by means of flow-cytometric DNA measurements. Expression of SC and IgA in neoplastic epithelium was positively related to differentiation in both groups. The AN tumours scored significantly higher for CEA than the ND ones, but the staining was apparently unrelated to differentiation or Dukes' stage. CEA expression in the transitional mucosa adjacent to ND tumours was negatively correlated with tumour differentiation, whereas epithelial IgA and SC in this zone showed a substantially higher positive correlation with tumour differentiation, and a somewhat stronger negative correlation with Dukes' stage in the ND than in the AN group. Plasma CEA levels were significantly related to Dukes' stage, only in patients with AN tumours, and only in this group were positively correlated with estimates of total tumour CEA for Dukes' stages A and B. For Dukes' stages C and D (disseminated tumours), moreover, the plasma CEA levels were found to be significantly higher in the AN group. These findings indicate that the DNA profile of large-bowel carcinomas is related both to the way neoplastic cells influence the activity of the transitional mucosa and their capacity for expression and release of epithelial products. AN tumours thus seem to be more active as "secretors" of CEA than ND ones.**

THE BEHAVIOUR of large-bowel carcinomas has hitherto been predicted mainly on the basis of clinico-pathological staging and histopathological grading. In addition, pre- and postoperative measurements of carcinoembryonic antigen (CEA) in plasma have shown prognostic value.

Another variable that may influence tumour behaviour is the cell content of nuclear DNA. Large-bowel carcinomas are composed of cell populations that are either near the diploid DNA level (2c) or show a higher nuclear DNA content (Enterline & Arvan, 1967; Böhm & Sandritter, 1975; Petersen *et al.*, 1978; Atkin & Kay, 1979). A "mosaic composition" of stem-cell lines with different nuclear DNA content has also been described (Stich & Steel, 1962; Petersen *et al.*, 1979; 1981). Conclusive prognostic indications have not emerged from such studies, however, though Atkin & Kay (1979) suggested that large-bowel carcinomas with near diploid or tetraploid levels may show slightly worse prognosis than tumours with other DNA profiles.

An additional recent line of research is immunohistochemical investigation of epithelial marker antigens. These include (*i*) functional characteristics of the normal intestinal epithelium, which may disappear partly or completely during malignant development, and (*ii*) carcinoembryonic antigens, which are present in foetal life and may reappear in neoplasia. Secretory IgA and secretory component (SC) belong to the first category. Thus, the functional role of SC as an epithelial receptor for dimeric IgA is well documented (Brandtzaeg, 1974, 1981; Brandtzaeg & Baklien, 1977; Brown, 1978) and previous studies have indicated that SC and epithelial IgA may be useful indicators of the degree of differentiation of neoplastic intestinal epithelium (Poger *et al.*, 1976; Weisz-Carrington *et al.*, 1976; Green *et al.*, 1977; Rognum *et al.*, 1980). The carcinoembryonic antigen (CEA) of Gold & Freedman (1965) represents the second type of epithelial marker. It has been demonstrated in the glycocalyx region of gastrointestinal epithelial cells (Gold *et al.*, 1968; von Kleist & Burtin, 1969*a*, *b*; Denk *et al.*, 1972) but its relation to malignancy is still a matter of controversy (Von Kleist & Burtin, 1969*a*, *b*; Denk *et al.*, 1972; Isaacson & Le Vann, 1976; Pihl *et al.*, 1980; Rognum *et al.*, 1980, 1982*a*, *b*; O'Brian *et al.*, 1981; Goslin *et al.*, 1981*a*).

Results of concurrent immunohistochemical staining of epithelial IgA and CEA have recently been reported for gastric malignancies (Ejeckam *et al.*, 1979) and for large-bowel carcinomas (Rognum *et al.*, 1980, submitted). In the latter investigations SC was also included, and the three markers were concurrently studied by paired immunofluorescence staining. In spite of large individual variations, it could be concluded that the expression of SC and IgA in neoplastic epithelial cells was positively correlated with the degree of tumour differentiation.

In the present study we evaluated the same three epithelial markers, and other variables such as plasma CEA levels, in relation to the DNA profile of large-bowel carcinomas. Our aim was to identify neoplastic cell populations with different biological properties. A minor part of this work has been presented in a preliminary communication (Rognum *et al.*, 1981).

### MATERIALS AND METHODS

*Tissue specimens.*—85 gross tumour specimens were obtained from 42 women and 41 men during surgery for adenocarcinoma of the large bowel. The mean age of the patients was 63·9 years (range 28–85). Further clinicopathological information appears in Tables I and II.

Immediately after resection, the tissue material was transported in ice-cold 0·01M phosphate buffer (pH 7·6) containing 0·15M NaCl (PBS) to the laboratory where the macro-pathological examination was done, and appropriate samples were excised for

TABLE I.—*Clinicopathological information about patients with near-diploid (ND) tumour-cell populations*

| Patient no. | Age (yrs) | Sex | Dukes' stage* | Differentiation (Evans)† | Plasma CEA (μg/l) |
|---|---|---|---|---|---|
| 1 | 60 | M | A | 3·5 | 3·6 |
| 2 | 73 | M | A | 4 | 3·4 |
| 3 | 80 | F | A | 3·5 | 4·9 |
| 4 | 64 | F | A | 4 | 1·5 |
| 5 | 65 | F | A | 4·5 | 0·5 |
| 6 | 76 | F | A | 5 | 0·8 |
| 7 | 47 | M | B | 3·5 | 3·7 |
| 8 | 81 | F | B | 2·5 | 672·0‡ |
| 9 | 80 | F | B | 3 | 310·0§ |
| 10 | 72 | F | B | 3 | 2·0 |
| 11 | 55 | M | B | 3·5 | 1·4 |
| 12 | 58 | F | B | 3 | 15·9 |
| 13 | 54 | M | B | 2·5 | 2·0 |
| 14 | 43 | M | B | 2·5 | 2·0 |
| 15 | 53 | F | B | 3·5 | 2·5 |
| 16 | 65 | F | B | 3 | 1·2 |
| 17 | 72 | F | B | 5 | 1·3 |
| 18 | 70 | F | B | 5 | 12·1 |
| 19 | 82 | F | C | 2·5 | 6·2 |
| 20 | 73 | F | C | 2 | 1·5 |
| 21 | 74 | M | C | 3 | 10·8 |
| 22 | 74 | F | C | 2·5 | 0·8 |
| 23 | 78 | F | C | 4 | 1·8 |
| 24 | 67 | F | C | 2 | 0·8 |
| 25 | 62 | F | C | 2 | 2·7 |
| 26 | 41 | F | D | 3 | 13·3 |
| 27 | 57 | F | D | 2 | 2·5 |
| 28 | 46 | M | D | 2·5 | 1·8 |

* Dukes & Bussey, (1958).

† Ashley (1978) (1 poorly differentiated, 5 well-differentiated.)

‡ The patient died postoperatively from sepsis.

§ The patient had a signet-ring-cell carcinoma strongly CEA+.

TABLE II.—*Clinicopathological information about patients with distinct aneuploid (AN) tumour-cell populations*

| Patient no. | Age (yrs) | Sex | Dukes' stage | Differentiation (Evans) | Approx. ploidy of heteroploid population | Plasma CEA (μg/l) |
|---|---|---|---|---|---|---|
| 1 | 73 | F | A | 2·5 | 3c | 1·2 |
| 2 | 65 | M | A | 4·5 | 3c, 6c, 7c | 1·8 |
| 3 | 58 | M | A | 2 | 5c | 1·8 |
| 4 | 76 | M | A | 4 | 4c | 3·0 |
| 5 | 40 | M | A | 4 | 4c | 2·1 |
| 6 | 64 | M | A | 4·5 | 4c | 3·7 |
| 7 | 74 | M | A | 5 | 4c | 0·8 |
| 8 | 28 | F | A | 3 | 4c | 1·4 |
| 9 | 58 | F | A | 1·5 | 4c | 5·5 |
| 10 | 52 | M | A | 3 | 3c | 0·8 |
| 11 | 63 | M | B | 4·5 | 4c | 2·8 |
| 12 | 60 | F | B<br>B | 2<br>2·5 | 3c<br>3c | 3·2 |
| 13 | 39 | F | B | 1 | 3c | 54·0 |
| 14 | 28 | F | B | 4 | 3c | 3·2 |
| 15 | 67 | M | B | 3 | 3c | 1·0 |
| 16 | 63 | F | B | 2·5 | 4c | 1·4 |
| 17 | 81 | M | B | 4 | 3c | 6·3 |
| 18 | 52 | M | B | 3 | 3c | 9·6 |
| 19 | 46 | F | B | 4·5 | 4c | 0·8 |
| 20 | 86 | M | B | 4 | 4c | 45·0 |
| 21 | 70 | M | B | 3 | 4c | 60·0 |
| 22 | 73 | M | B | 3 | 4c | 0·8 |
| 23 | 69 | M | B<br>B | 3<br>4 | 4c<br>4c | 5·5 |
| 24 | 66 | M | B | 4 | 3c | 7·4 |
| 25 | 76 | M | B | 3·5 | 3c | 5·1 |
| 26 | 69 | M | B | 3·5 | 3c | 9·0 |
| 27 | 83 | M | B | 2·5 | 4c | 0·8 |
| 23 | 69 | M | B | 4 | 4c | |
| 24 | 66 | M | B | 4 | 3c | 7·4 |
| 25 | 76 | M | B | 3·5 | 3c | 5·1 |
| 26 | 69 | M | B | 3·5 | 3c | 9·0 |
| 27 | 83 | M | B | 2·5 | 4c | 0·8 |
| 28 | 78 | F | B | 3 | 4c | 4·6 |
| 29 | 63 | F | B | 5 | 4c | 2·5 |
| 30 | 6£ | M | B | 3 | 4c | 3·1 |
| 31 | 81 | F | C | 2·5 | 3c | 10·3 |
| 32 | 56 | M | C | 1·5 | 4c | 0·8 |
| 33 | 67 | F | C | 3 | 3c | 4·6 |
| 34 | 55 | F | C | 3·5 | 3c | 13·7 |
| 35 | 77 | F | C | 2 | 4c | 3·9 |
| 36 | 40 | M | C | 1·5 | 4c | 2·1 |
| 37 | 68 | F | C | 3 | 3c | 1·8 |
| 38 | 70 | F | C | 4 | 3c | 11·4 |
| 39 | 35 | M | C | 4 | 3c | 3·1 |
| 40 | 76 | F | C | 3 | 3c | 4·9 |
| 41 | 69 | F | C | 3 | 4c | 159·0 |
| 42 | 68 | M | C | 3 | 3c, 4c | 4·1 |
| 43 | 45 | F | C | 1 | 3c | 0·8 |
| 44 | 56 | F | C | 2 | 3c | 2·5 |
| 45 | 69 | M | D | 1 | 3c | 750·0 |
| 46 | 78 | M | D | 3 | 3c, 4c | 161·0 |
| 47 | 77 | M | D<br>D | 2<br>2 | 3c, 4c<br>3c | 635·0 |
| 48 | 80 | F | D | 3 | 4c | 100·0 |
| 49 | 73 | M | D | 2 | 4c | 91·0 |
| 50 | 85 | M | D | 4 | 3c | 9·5 |
| 51 | 55 | F | D | 2·5 | 4c | 5·8 |
| 52 | 44 | M | D | 3 | 3c | 0·8 |
| 53 | 68 | M | D | 3 | 3c | 7·5 |
| 54 | 40 | F | D | 3 | 3c | 11·5 |

DNA measurement and immunohistochemistry.

*Flow cytometry.*—In 60 cases, 4 samples from the tumour edge and 1 from the centre were subjected to DNA quantitation by flow cytometry (FCM). For the remaining 25 specimens less than 5 samples from each tumour were examined. Single-cell suspensions were prepared immediately after sample excision by mincing the tissue in PBS, followed by filtration through a nylon mesh (pore size 70 $\mu$m). The cells were fixed in ice-cold absolute ethanol and kept in 70% ethanol until processed for FCM by exposure to RNase (Boehring, Mannheim, FRG; 1 g/l in water, 1 h, 37°C) and pepsin (Orthana, Copenhagen, Denmark; 4 g/l in 0·02N HCl, 15 min, 37°C) prior to staining according to Göhde & Dittrich (1971) with ethidium bromide (Calbiochem, California, U.S.A.). Emission measurements were performed in an ICP 11 flow cytometer (PHYWE AG, Göttingen, FRG).

The FCM histograms were analysed by planimetry (Göhde, 1973), and the percentages of pulses over the diploid level were calculated. Mouse spleen lymphocytes were used as a diploid (2c) reference, and peaks within 25% of this standard were assigned to near-diploid (ND). One or more distinct peaks occurring above that level were regarded aneuploid (AN). Minor 4c peaks might represent $G_2$ cells of a diploid cell population, $G_1$ cells of a tetraploid population, or a combination of both. When the area under the 4c peak was greater than that between the 2c and 4c levels, the 4c peak was taken to represent an AN cell population. This generally seemed justifiable, since, in proliferating mammalian cell populations, the proportion of cells in $G_2$ is generally lower than that in S, (Steel, 1977). However, additional interpretative problems were occasionally caused by cell clumping, which was assumed to be reflected in a 6c peak; when its area exceeded 10% of the 4c peak the latter was not accepted for assignment to distinct AN. Such tumours were assigned to the ND group, and added to its apparent heterogeneity.

To estimate the content of non-epithelial cells in the suspensions, smears were stained with May–Grünwald–Giemsa stain. The proportions of granulocytes and small mononuclear cells were counted by light microscopy in 55 samples from 14 tumours.

*Immunohistochemistry.*—The immunohistochemical investigation was in every tumour based on two tissue samples, both containing neoplastic epithelium and adjacent transitional mucosa. As controls, 47 histologically normal samples of large-bowel mucosa were included. After direct ethanol fixation, the samples were processed for paraffin embedding (Brandtzaeg, 1974).

Serial sections, cut at 6 $\mu$m from each tissue block, were subjected to paired immunofluorescence staining. Fluorescein isothiocyanate (FITC)-labelled sheep anti-SC was combined with tetramethylrhodamine isothiocyanate (MRITC)-labelled rabbit anti-CEA; and FITC-labelled rabbit anti-IgA was combined with MRITC-labelled sheep anti-SC. The preparation and characterization of these sheep and rabbit IgG-fluorochrome conjugates have been reported previously (Rognum *et al.*, 1980). A third adjacent section was stained with a trichrome routine method combining haematoxylin, azofloxine and saffron (Stave & Brandtzaeg, 1977). The Leitz Orthoplan fluorescence microscope was equipped with an Osram HBO 200 W lamp for excitation of rhodamine (red) emission, and with an XBO 150 W lamp for fluorescein (green). Narrow-band excitation and selective filtration of the fluorescence colours were obtained with a Ploem-type epi-illuminator.

The fluorescence intensity of the three epithelial marker antigens (CEA, SC, and IgA) was evaluated with the semiquantitative scoring system detailed elsewhere (Rognum *et al.*, 1980). Briefly, a score of 3 indicated strong intensity and 0 negligible or non-staining. For each marker, every specimen was assigned an average score based on the evaluation of two tissue samples. The same investigator was responsible for this evaluation throughout the investigation; a blind study of reproducibility revealed no systematic error (Rognum *et al.*, 1980).

*Histopathological grading of tumour differentiation.*—Sections stained for conventional histopathology were randomized and graded using the criteria of Evans (Ashley, 1978); a score of 1 referring to poorly differentiated carcinoma and 5 indicating high differentiation. The same pathologist performed all this grading, and a blind reproducibility control revealed no systematic error (Rognum *et al.*, 1980).

*Clinico-pathological tumour staging.*—The extent of bowel involvement and the presence or absence of lymph-node metastasis were staged in accordance with the criteria given by Dukes & Bussey (1958). In addition to Dukes' stages A, B, and C, tumours with distant organ metastasis were assigned to stage D.

*Estimation of total tumour CEA.*—A volumetric estimate of all localized tumours (Dukes' stage A and B) was based on the most appropriate geometric formula and size measurements of the gross specimen. This estimate was multiplied by the anti-logarithm of the fluorescence CEA score of the tumour to obtain a relative expression of its total CEA content. It has been shown that immunofluorescence staining intensity is linearly correlated with the logarithm of the antigen concentration (Brandtzaeg, 1972).

*Plasma CEA levels.*—The concentration of CEA in plasma was quantified by radioimmunoassay with a slightly modified CEA-Roche test (Ørjasaeter *et al.*, 1978).

*Statistical methods.*—Since our observations were mainly expressed as scores, grades or stages, only statistical methods that are compatible with the application of the ordinal scale were considered valid (Stevens, 1946). Non-parametric rank methods were therefore used, and distributions were given as medians and observed ranges. Correlations were based on Kendall's test, and group comparisons were made by the Mann–Whitney U test (Siegel, 1956; Fenstad *et al.*, 1977).

### RESULTS

### *Flow-cytometric DNA quantitation*

On the basis of the DNA profile, 28 of the tumours were assigned to the near diploid (ND) group (Fig. 1); the remaining 57 could be defined as distinctly aneuploid (AN) but all of them contained in addition ND cells (Fig. 2).

Conventional May–Grünwald–Giemsa staining of smeared cell suspensions indicated that the contamination with small mononuclear cells and granulocytes was < 5% in 50/55 samples and < 20% in the remaining 5. Immunofluorescence staining of dispersed cells showed expression of marker proteins in concordance with results from tissue sections of the same tumour (Fig. 3).

FIG. 1.—DNA histogram from a tumour with no aneuploid cell population (i.e. near-diploid). Level of mouse lymphocyte fluorescence indicated by arrow.

FIG. 2.—DNA histogram from a tumour containing a distinctly aneuploid cell population. Level of mouse lymphocyte fluorescence indicated by arrow.

### *Intra-individual variations in DNA pattern*

In 60 specimens (from which 5 tumour samples were subjected to flow cytometry) no discrepancies were seen between DNA profiles, and hence assignment to ploidy group. However, in the AN group, 4 cases showed more than one peak above diploid (Table II). No statistically signifi-

FIG. 3.—Dissociated cell from a moderately to well-differentiated AN tumour stained with MRITC-labelled anti-CEA (a) and with FITC-labelled anti-SC (b). The red fluorescence for CEA is confined to the apex of the cell, whereas the green fluorescence for SC is diffusely distributed throughout the cytoplasm. × 920.

cant difference was revealed in percentage of pulses above diploid ($P > 0{\cdot}5$) when the tumour edge (medians of 4 samples) was compared with the tumour centre (one sample) in 60 specimens.

*General staining characteristics of the epithelial markers*

When the neoplastic epithelium contained detectable cell markers, they were generally distributed evenly throughout the tumour. If only scattered positive epithelial elements were seen in an otherwise negative tumour, such areas were disregarded in assigning fluorescence scores. In the whole material, scores for SC and IgA were positively correlated with degree of tumour differentiation ($\tau = 0{\cdot}30, P < 0{\cdot}01$ for both) whereas CEA expression showed no relationship to tumour differentiation. Nevertheless, CEA scores for neoplastic epithelium were significantly higher than for normal mucosa ($P < 0{\cdot}01$) regardless of tumour ploidy (Fig. 6). SC and IgA staining showed the opposite result (Fig. 6) and there was an inverse relationship between tumour CEA and tumour SC or IgA ($\tau = -0{\cdot}30$, $P < 0{\cdot}01$).

$SC^+$ tumours usually exhibited diffuse intracellular staining, but apical intensification was noted in glandular elements. Epithelial staining for IgA was generally confined to the apices of the cells. Accumulations within glandular lumina were sometimes positive for both SC and IgA.

CEA staining was mainly confined to a rim lining the apices of the tumour cells, but the fluorescence was usually intensified by intercellular extensions, and often by diffuse cytoplasmic staining (Fig. 4). $CEA^+$ material was commonly seen in lumina of gland-forming elements. Intratumour variations in CEA staining never exceeded one score (Rognum *et al.*, 1980) and abrupt changes between bright and negative areas were not seen.

The contents of goblet-like cells in well-differentiated carcinomas were always negative for SC, IgA, and CEA (Fig. 5). However, in a case of signet-ring-cell carcinoma the neoplastic cells contained all 3 markers.

In the transitional mucosa adjacent to the tumour all 3 epithelial markers where distincly localized to the columnar cells, and the mucin content of goblet cells was unstained (Figs 4 & 5). Some heterogeneity with regard to staining intensity was noted, as reported previously (Rognum *et al.*, 1980); each final score was, therefore, an average for two mucosal samples from the same specimen. When hyperplastic crypt elements were present, staining for CEA was generally intense, whereas that for SC and epithelial IgA was faint. In the whole material, CEA scores were

FIG. 4.—Poorly differentiated large-bowel carcinoma. (a) Routine staining; frame indicates area studied immunohistochemically. (b, c, d) Adjacent sections incubated with MRITC-labelled anti-CEA (b) FITC-labelled anti-SC (c) and FITC-labelled anti-IgA (d). CEA abundant both in the transitional mucosa (Tr) and in the tumour (Tu), whereas SC and IgA were present in normal amounts in the transitional mucosa, but lacking in the tumour. Goblet cells appeared unstained. (a) × 19, (b) (c) and (d) × 113.

FIG. 5.—Well-differentiated large-bowel carcinoma. (a) Routine staining; frame indicates area studied immunohistochemically. (b, c, d) Adjacent sections incubated with MRITC-labelled anti-CEA (b) FITC-labelled anti-SC (c) and FITC-labelled anti-IgA (d). CEA, SC, and epithelial IgA were present both in the transitional mucosa (Tr) and in the tumour (Tu) but not in the mucin-containing elements. (a) × 19, (b) (c) and (d) × 115.

significantly higher in the transitional mucosa than in normal epithelium ($P < 0{\cdot}02$) whereas the reverse was true for both SC and IgA scores ($P < 0{\cdot}01$) (Fig. 7).

*Immunohistochemical staining results in relation to tumour DNA ploidy*

Tumour CEA scores were significantly higher ($P < 0{\cdot}02$) in AN than in ND tumours (Fig. 6). Conversely, there was only a trend for the AN tumours to show lower SC ($P \sim 0{\cdot}14$) and IgA ($P \sim 0{\cdot}06$) scores than the ND ones (Fig. 6).

In the transitional mucosa, staining for the 3 epithelial markers revealed no significant differences between the 2

FIG. 6.—Immunofluorescence scores for aneuploid (●) or near diploid (○) adenocarcinomas compared with scores for histologically normal epithelium of control samples (△). Medians are indicated by horizontal lines. For both tumour groups considered together, CEA scores were significantly higher than for controls ($P < 0{\cdot}01$), whereas SC and IgA scores were lower ($P < 0{\cdot}001$). Aneuploid tumours had moreover, significantly higher CEA scores than near-diploid tumours ($P < 0{\cdot}02$) whereas the latter tended to have higher SC and IgA scores.

tumour ploidies (Fig. 7). However, in the ND group, SC and IgA scores showed positive correlation with the degree of tumour differentiation ($\tau = 0{\cdot}59$, and $\tau = 0{\cdot}52$, respectively; $P < 0{\cdot}01$) and an inverse relationship to Dukes' stage ($\tau = -0{\cdot}44$ and $\tau = -0{\cdot}39$, respectively; $P < 0{\cdot}01$). Only weak similar trends were seen in the AN group (Figs 8 & 9, Table III). In the ND group, moreover, a negative correlation appeared between CEA scores in the transitional mucosa and the degree of tumour differentiation ($\tau = -0{\cdot}43$, $P < 0{\cdot}01$) whereas the reverse seemed to be true in relation to Dukes' stage ($\tau = 0{\cdot}32$, $P < 0{\cdot}02$).

*Degree of tumour differentiation, Dukes' stage, and plasma CEA level in relation to tumour ploidy*

The distribution of degrees of tumour differentiation and Dukes' stages seemed to be similar for the two ploidy groups. For the whole material, Dukes' stage was negatively correlated with tumour differentiation ($\tau = -0{\cdot}38$, $P < 0{\cdot}01$) and this correlation was better in the ND ($\tau = -0{\cdot}58$, $P < 0{\cdot}001$) than in the AN group ($\tau = -0{\cdot}30, P < 0{\cdot}01$).

No clear relationship between plasma CEA level and tumour differentiation was seen in the material.

However, the CEA level was positively correlated with Dukes' stage and this correlation was slightly better in the AN group ($\tau = 0{\cdot}39, P < 0{\cdot}01$) than in the whole material ($\tau = 0{\cdot}28$, $P < 0{\cdot}01$); by contrast, no such relationship appeared in the ND group (Fig. 10). In addition, the plasma CEA level tended to be higher in the AN than in the ND group ($P \sim 0{\cdot}09$), and this trend was mainly accounted for by the difference ($P \sim 0{\cdot}05$) between tumours of Dukes' stages C and D (Fig. 10).

FIG. 7.—Immunofluorescence scores for epithelium of transitional mucosa adjacent to aneuploid (●) or near diploid (○) adenocarcinomas compared with scores for histologically normal epithelium of control samples (△). Medians are indicated by horizontal lines. For both tumour groups, CEA scores were significantly higher in the transitional mucosa than in the controls ($P < 0{\cdot}02$), whereas SC- and IgA scores in this zone were lower ($P < 0{\cdot}01$). There were no significant differences between the ploidy groups.

*Plasma CEA level in relation to total tumour CEA*

In the AN group the plasma CEA level showed a significant positive correlation ($\tau = 0{\cdot}33$, $P < 0{\cdot}02$) with the estimated total CEA content of localized tumours (Dukes' stage A and B) (Fig. 11). This result was not found for either localized tumours in the ND group, or when the material was considered as a whole for Dukes' stage A and B; even on selecting tumours with CEA scores of at least 1·75.

### DISCUSSION

Characterization of large-bowel carcinomas in prognostic terms are currently based on clinicopathological staging and histological grading of tumour differentiation. Plasma CEA measurements may afford additional prognostic information and be of particular value in follow-up studies after surgery (Mach *et al.*, 1974; Zamcheck *et al.*, 1975; Meeker, 1978; Lavin *et al.*, 1981; Wanebo *et al.*, 1981; N.I.H., 1981). Furthermore, some attempts have been made to characterize large-bowel carcinomas by applying epithelial cell-differentiation markers and by measuring nuclear DNA. However, the biological significance of these methods has not been sufficiently established.

Previous studies of nuclear DNA in tumours, using Feulgen absorption cytophotometry, have indicated that aneuploidy and increased amounts of DNA are related to the degree of malignancy (Böhm & Sandritter, 1975; Atkin & Kay, 1979). The development of flow cytometry (FCM) and its application to cell suspensions from solid tumours makes it possible to measure DNA profiles without time-consuming manual focusing on each cell (for review, see Laerum & Farsund, 1981). The DNA profiles obtained by Feulgen microspectro-photometry in mammary

FIG. 8.—Scatter diagrams of relations between degree of tumour differentiation and transitional mucosa SC score in near diploid (a) and aneuploid (b) tumours.

FIG. 9.—Scatter diagrams of relations between Dukes' stage and transitional mucosa SC score in near diploid (a) and aneuploid (b) tumours.

carcinomas correlate well with those obtained by flow cytometry (Auer & Tribukait, 1980), allowing direct comparison between results from the 2 methods.

It seemed appropriate to divide our large-bowel carcinomas into a group with a near-diploid nuclear DNA content (< 2·5c) and another also containing one or more distinctly aneuploid cell populations (> 2·5c). It is well known that solid tumours may contain considerable numbers of macrophages (Evans, 1973) but a recent study has shown that in most colonic carcinomas few such cells occur within the tumour, most of them being at the periphery (Svennevig, 1980). Granulocytes and small mononuclear cells, which presumably would initiate near-diploid pulses in FCM, usually accounted for < 5% of our cell suspensions, and could not therefore account for the near-diploid peaks seen in the AN tumours. In an FCM study of 6 large-bowel carcinomas, Petersen *et al.* (1979) similarly concluded that contamination with normal cells was much too low to explain the frequency of cells with a near-normal DNA content. Thus, AN cell populations apparently contain a proportion of near-diploid neoplastic cells. The mosaic constitution of colonic carcinomas, as indicated by the presence in most tumours

Fig. 10.—Scatter diagrams of relations between Dukes' stage and plasma CEA level. The median of the plasma CEA levels within each stage is indicated by horizontal line. The plasma CEA levels in cases of Dukes' stages C and D were significantly higher in the aneuploid than in the near-diploid group ($P \sim 0{\cdot}05$). The plasma CEA levels were, moreover, positively correlated with the Dukes' stage in the aneuploid ($\tau = 0{\cdot}39$, $P < 0{\cdot}01$) but not in the near-diploid group ($\tau = 0{\cdot}03$, $P < 0{\cdot}8$).

Fig. 11.—Scatter diagram of relation between estimates for total tumour CEA (tumour volume multiplied by antilogarithm of CEA score) and plasma CEA level in patients with localized aneuploid tumours (Dukes' stages A and B).

Table III.—*Relationships of epithelial SC and IgA to degree of tumour differentiation, Dukes' stage, and plasma CEA level (examined by Kendall's τ test)*

| | Tumour | | Transitional mucosa | |
|---|---|---|---|---|
| Pair of variables | τ | *P* | τ | *P* |
| All tumours n = 85 | | | | |
| SC *vs* differentiation | 0·30 | <0·01 | 0·29 | <0·01 |
| SC *vs* Dukes' stage | −0·18 | <0·02 | −0·27 | <0·01 |
| SC *vs* plasma CEA | −0·24 | <0·01 | −0·09 | n.s. |
| IgA *vs* differentiation | 0·30 | <0·01 | 0·31 | <0·01 |
| IgA *vs* Dukes' stage | −0·18 | <0·05 | −0·27 | <0·01 |
| IgA *vs* plasma CEA | −0·27 | <0·01 | −0·09 | n.s. |
| Aneuploid n = 57 | | | | |
| SC *vs* differentiation | 0·33 | <0·01 | 0·19 | <0·05 |
| SC *vs* Dukes' stage | −0·16 | n.s. | −0·19 | <0·05 |
| SC *vs* plasma CEA | −0·11 | n.s. | −0·04 | n.s. |
| IgA *vs* differentiation | 0·37 | <0·01 | 0·27 | <0·01 |
| IgA *vs* Dukes' stage | −0·18 | <0·05 | −0·20 | <0·05 |
| IgA *vs* plasma CEA | −0·26 | <0·01 | 0·02 | n.s. |
| Near diploid n = 28 | | | | |
| SC *vs* differentiation | 0·30 | <0·05 | 0·59 | <0·01 |
| SC *vs* Dukes' stage | −0·20 | n.s. | −0·44 | <0·01 |
| SC *vs* plasma CEA | −0·19 | n.s. | −0·14 | n.s. |
| IgA *vs* differentiation | 0·33 | <0.01 | 0·52 | <0·01 |
| IgA *vs* Dukes' stage | −0·09 | n.s. | −0·39 | <0·01 |
| IgA *vs* plasma CEA | −0·29 | <0·05 | −0·31 | <0·01 |

of more than one cell clone with distinctive DNA content, is in agreement with the recent results of Petersen *et al.* (1981). Clonal heterogeneity is further substantiated by the highly variable proportions of the different clones occurring within individual tumours (Petersen *et al.*, 1981; Rognum *et al.*, 1981).

We found no significant difference in the percentage of pulses above 2·5c between samples from the tumour edge and the centre in 60 tumours; a result agreeing with the observation of Böhm & Sandritter (1975). Thus, environmental conditions at the edge do not seem to produce a systematic predominance of normal-cell admixture (diploid cells).

In accordance with others (Wagener *et al.*, 1981) we generally found that CEA was evenly distributed within individual tumours. Conversely, large variations in staining intensity were seen *between* tumours; many AN ones containing more CEA than the ND ones. In mammary carcinomas Wittekind *et al.* (unpublished) found no obvious correlation between CEA staining and DNA profile, which, however, was based on other criteria than those used in our study. According to our experience, moreover, mammary carcinomas show relatively heterogeneous CEA expression (unpublished observations); direct comparison with large-bowel carcinomas may not, therefore, be justified.

Earlier reports have related CEA staining of large-bowel carcinomas, or the concentration of extractable tumour CEA, to the degree of tumour differentiation and other morphological variables, such as growth pattern, tumour necrosis, and vessel invasion. Denk *et al.* (1972) concluded that well-differentiated carcinomas contained larger amounts of CEA than anaplastic ones, whereas most others have been unable to confirm such a relationship (Bordes *et al.*, 1973; Pihl *et al.*, 1980; Rognum *et al.*, 1980; Wagener *et al.*, 1981). Conversely, a positive correlation between the amount of both tumour SC and epithelial IgA and the degree of differentiation has emerged from several previous reports (Poger *et al.*, 1976; Weisz-Carrington *et al.*, 1976; Green *et al.*, 1977; Rognum *et al.*, 1980) and we found that the AN tumours tended to express less of both markers than ND ones. The indication of an inverse relationship between tumour CEA and tumour SC or IgA in our material, suggests that application of different types of markers may be complementary and hence of enhanced value for evaluation of adenocarcinomas, as recently discussed also by Ejeckam *et al.* (1979).

As reported previously (Rognum *et al.*, 1980) staining for both SC and epithelial IgA in the transitional mucosa was positively correlated with the degree of tumour differentiation, and inversely related to Dukes' stage; these relationships turned out to be particularly well expressed in the ND group of tumours. This also held true for the negative relationship between CEA in this zone and tumour differentiation. Thus, the less well-differentiated ND tumours seemed to affect adversely SC production and to induce more CEA in the adjacent epithelium than the well-differentiated ones; similar trends were apparent in relation to Dukes' stage. Microenvironmental influences of a tumour hence seem to depend on both differentiation and ploidy in an as yet unexplained manner.

We found only a weak positive correlation between the preoperative plasma CEA level and Dukes' stage in the material as a whole, but this correlation was substantially better in the AN group of tumours. The plasma CEA levels were, moreover, significantly higher in patients with disseminated AN tumours. Thus, it seems that differences in DNA profiles of various tumour materials may at least partly account for the present disagreement over whether plasma CEA levels are related to tumour load.

Our study does not definitely support the view that poorly differentiated tumour are associated with particularly high plasma CEA levels (Zamcheck *et al.*, 1975; Pihl *et al.*, 1980). Also the suggestion that such tumours tend to release relatively little CEA (Goslin *et al.*, 1981b; N.I.H., 1981) needs further investigation (Rognum *et al.*, submitted). We favour the hypothesis that large-bowel carcinomas may be classified as "secretors" or "non-secretors" of CEA (von Kleist *et al.*, unpublished) and that this characteristic is related to the DNA profile of the neoplastic cells (Wittekind *et al.*, unpublished). A positive relationship thus appeared between estimated total tumour CEA and plasma CEA level only in patients with localized AN tumours. This result could not be ascribed merely to a higher CEA content of the AN tumours, but apparently reflected a proneness to release CEA into the circulation. Moreover, as mentioned above, a relationship between Dukes' stage and plasma CEA level was only found in patients with AN tumours.

In conclusion, the ability of tumour cells to express SC, and thereby to retain the capacity for uptake of dimeric IgA, may be taken as a sign of differentiation. Accordingly, carcinomas with this function largely intact are generally well-differentiated. Conversely, increased expression of CEA by neoplastic cells cannot simply be considered as a sign of tumour immaturity. Semiquantitative tumour CEA estimation combined with FCM determination of DNA ploidy may, however, turn out to be a useful way of selecting patients who should be followed-up by repeated plasma CEA measurements. Clinical follow-up of our patients will show whether the above-discussed ways of assessing biological characteristics of large-bowel carcinomas are of prognostic value.

The study was supported by the Norwegian Cancer Society, Ole and Rolf Norberg's Legacy, and Anders Jahre's Foundation. The authors are grateful to Gunnvor Øijordsbakken, Aasa Schjølberg and Gunn Jamne for skilled technical assistance, and to Elisabeth Falleth for help with data processing.

### REFERENCES

ASHLEY, J. B. (1978) *Evan's Histological Appearance of Tumours*, 3rd edn. Edinburgh: Livingstone. p. 582.

ATKIN, N. B. & KAY, R. (1979) Prognostic significance of modal DNA value and other factors in malignant tumors, based on 1465 cases. *Br. J. Cancer*, **40**, 210.

AUER, G. & TRIBUKAIT, B. (1980) Comparative single cell and flow DNA analysis in aspiration biopsies from breast carcinomas. *Acta Pathol. Microbiol. Scand. Sect. A*, **88**, 355.

BORDES, M., MICHIELS, R. & MARTIN, F. (1973) Detection by immunofluorescence of carcionembryonic antigen in colonic carcinoma, other malignant or benign tumours, and non-cancerous tissues. *Digestion*, **9**, 106.

BRANDTZAEG, P. (1972) Evaluation of immunofluorescence with artificial sections of selected antigenicity. *Immunology*, **22**, 177.

BRANDTZAEG, P. (1974) Mucosal and glandular distribution of immunoglobulin components: Immunohistochemistry with a cold ethanol fixation technique. *Immunology*, **26**, 1101.

BRANDTZAEG, P. & BAKLIEN, K. (1977) Intestinal secretion of IgA and IgM: A hypothetical model. In *Immunology of the Gut*. CIBA Foundation Symposium, **46**, Amsterdam: Elsevier North-Holland. p. 77.

BRANDTZAEG, P. (1981) Transport models for secretory IgA and secretory IgM. *Clin. Exp. Immunol.*, **44**, 221.

BÖHM, N. & SANDRITTER, W. (1975) DNA in Human Tumors: A cytophotometric study. *Current Top. Pathol.* **60**, 151.

BROWN, W. R. (1978) Relationship between immunoglobulins and the intestinal epithelium. *Gastroenterology*, **75**, 129.

DENK, H., TAPPEINER, G., ECKERSDORFER, R. & HOLZNER, J. H. (1972) Carcinoembryonic antigen (CEA) in gastrointestinal and extra-gastrointestinal tumours and its relationship to tumour-cell differentiation. *Int. J. Cancer*, **10**, 262.

DUKES, C. E. & BUSSEY, H. J. R. (1958) The spread of rectal cancer and its effect on prognosis. *Br. J. Cancer*, **12**, 309.

EJECKAM, G. C., HUANG, S. N., MCCAUGHEY W. T. E. & GOLD, P. (1979) Immunohistopathologic study on carcinoembryonic antigen (CEA)-like material and immunoglobulin A in gastric malignancies. *Cancer*, **44**, 1606.

ENTERLINE, H. T. & ARVAN, D. A. (1967) Chromosome constitution of adenoma and adenocarcinoma of the colon. *Cancer*, **20**, 1746.

EVANS, R. (1973) Macrophages and the tumour bearing host. *Br. J. Cancer*, **28**, Suppl. I, 19.

FENSTAD, G. U., WALLÖE, L. & WILLE, Ø. (1977) Three tests for regression compared by stochastic simulation under normal and heavy tailed distribution of errors. *Scand. J. Statist.* **4**, 31.

GÖHDE, W. (1973) *Zellzyklusanalysen mit dem Impulscytophotometer: Der Einfluss chemischer und physikalischer Noxen auf Prolieferationskinetik von Tumorzellen*. Münster: Thesis.

GÖHDE, W. & DITTRICH, W. (1971) Impulsfluorometrie, ein neuartiges Durchflussverfahern zur ultraschnellen Mengenbestimmung von Zellinhaltstoffen. *Acta Histochem.* (Suppl) **10**, 429.

GOLD, T., GOLD, M. & FREEDMAN, S. O. (1968) Cellular location of carcinoembryonic antigens of the human digestive system. *Cancer Res.*, **28**, 1331.

GOLD, P. & FREEDMAN, S. O. (1965) Specific carcinoembryonic antigens of the human digestive system. *J. Exp. Med.*, **122**, 467.

GOSLIN, R., STEELE, G., MACINTYRE, J. & 5 others (1981*a*) The use of preoperative plasma CEA levels for the stratification of patients after curative resection of colorectal cancers. *Ann. Surg.* **192**, 747.

GOSLIN R., O'BRIAN, M. J., STEELE, G., MAYER, R., WILSON, R., CORSON, J. M. & ZAMCHECK, N. (1981b) Correlation of plasma CEA and CEA tissue staining in poorly differentiated colorectal cancer. *Am. J. Med.* **71**, 246.

GREEN, F. H. Y., WHITEHEAD, S. & FOX, H. (1977) Abnormalities of the local immune system in intestinal neoplasia: A morphological study. *J. Pathol.*, **122**, 55.

ISAACSON, P. & LE VANN, H. P. (1976) The demonstration of carcinoembryonic antigen in colorectal carcinoma and colonic polyps using an immunoperoxidase technique. *Cancer*, **38**, 1348.

LAERUM, O. D. & FARSUND, T. (1981) Clinical application of flow cytometry. *Cytometry*, **2**, 1.

LAVIN, P. T., DAY, J. HOLYOKE, E. D., MITTLEMAN, H. & SHU, T. M. (1981) A statistical evaluation of baseline and follow-up carcinoembryonic antigen in patients with resectable colorectal carcinoma. *Cancer*, **47**, 823.

MACH, J. P., JAEGER, P., BERTHOLET, M. M., RUESEGGER, C. H., LOOSLI, & PETTAVEL, J. (1974) Detection of recurrence of large-bowel carcinoma by radioimmunoassay of circulating carcinoembryonic antigen (CEA). *Lancet*, ii, 535.

MEEKER, W. R. (1978) Use and abuse of CEA test in clinical practice. *Cancer*, **41**, 854.

NATIONAL INSTITUTES OF HEALTH CONSENSUS DEVELOPMENT CONFERENCE STATEMENT (1981) Carcinoembryonic antigen: Its role as a marker in the management of cancer. *Cancer Res.* **41**, 2017.

O'BRIAN, M. J., ZAMCHECK, N., BURKE, B., KIRKHAM, S. E., SARAVIS, C. A. & GOTTLIEB, L. S. (1981) Immunocytochemical localization of carcinoembryonic antigen in benign and malignant colorectal tissues. *Am. J. Clin. Pathol.* **75**, 283.

ØRJASAETER, H., FOSSÅ, S. D., SCHJOESETH, S. A. & FJAERSTAD, K. (1978) Carcinoembryonic antigen (CEA) in plasma of patients with carcinoma of the bladder/urethra. *Cancer*, **42**, 287.

PETERSEN, W. E., BICHEL, P. & LORENTZEN, M. (1979) Flow-cytometric demonstration of tumor cell subpopulations with different DNA content in human colo-rectal carcinoma. *Eur. J. Cancer*, **15**, 383.

PETERSEN, S. E., LORENTZEN, H. & BICHEL, P. (1981) A mosaic subpopulation structure of human colorectal carcinomas demonstrated by flow cytometry. *Acta Pathol. Scand. A* (Suppl) **274**, 412.

PIHL, E., MCNAUGHTAN, J., MA, J., WARD, H. A. & NAIRN, R. C. (1980) Immunohistological patterns of carcinoembryonic antigen in colorectal carcinoma: Correlation with staging and blood levels. *Pathology*, **12**, 7.

POGER, M. E., HIRSCH, B. R. & LAMM, M. E. (1976) Synthesis of secretory component by colonic neoplasms. *Am. J. Pathol.*, **82**, 327.

ROGNUM, T. O., BRANDTZAEG, P., ØRJASAETER, H., ELGJO, K. & HOGNESTAD, J. (1980) Immunohistochemical study of secretory component, secretory IgA and carcinoembryonic antigen in large bowel carcinomas. *Pathol. Res. Pract.*, **170**, 126.

ROGNUM, T. O., ELGJO, K., FAUSA, O. & BRANDTZAEG, P. (1982b) Immunohistochemical evaluation of carcinoembryonic antigen, secretory component, and epithelial IgA in ulcerative colitis with dysplasia. *Gut*, **23**, 123.

ROGNUM, T. O., FAUSA, O. & BRANDTZAEG, P. (1982a) Immunohistochemical evaluation of carcinoembryonic antigen, secretory component and epithelial IgA in tubular and villous large bowel adenomas with different grades of dysplasia. *Scand. J. Gastroenterol.*, **17**, 341.

ROGNUM, T. O., THORUD, E., ELGJO, K., & 4 others (1981) DNA flow cytometry (FCM) in carcinomas of the large bowel compared with the functional cell markers secretory component (SC) and carcinoembryonic antigen (CEA), the histological tumour grade, and the clinical stage. A preliminary communication. *Acta Pathol. Microbiol. Scand. Sect. A* (Suppl.) **274**, 417.

SIEGEL, S. (1956) *Non-parametric Statistics for the Behavioral Sciences.* Tokyo: McGraw-Hill Kogakuska. p. 223.

STAVE, R. & BRANDTZAEG, P. (1977) Fluorescence staining pattern of gastric mucosa: A study with special reference to parietal cells. *Scand. J. Gastroenterol.*, **12**, 885.

STEEL, G. G. (1977) *Growth kinetics of tumours.* Oxford: Clarendon Press. p. 202.

STEVENS, S. S. (1946) On the theory of scales of measurement. *Science*, **103**, 677.

STICH, H. F. & STEELE, H. D. (1962) DNA content of tumor cells III. Mosaic composition of sarcomas and carcinomas in man. *J. Natl Cancer Inst.*, **28**, 1207.

SVENNEVIG, J. L. (1980) *In situ* identification of inflammatory cells in malignant, non-lymphoid human tumours. *Acta Pathol. Microbiol. Scand. Sect. A*, **88**, 387.

VON KLEIST, S. & BURTIN, P. (1969*a*) Isolation of a fetal antigen from human colonic tumors. *Cancer Res.*, **29**, 1961.

VON KLEIST, S. & BURTIN, P. (1969*b*) Localisation cellulaire d'un antigene embryonaire de tumeurs colique humaines. *Int. J. Cancer*, **4**, 874.

WAGENER, C., MÜLLER-WALLRAF, R., NISSON, S., GRÖNER, J. & BREVER, H. (1981) Localization and concentration of carcinoembryonic antigen in gastrointestinal tumors: Correlation with CEA levels in plasma. *J. Natl Cancer Inst.* **67**, 539.

WANEBO, H. J. (1981) Are carcinoembryonic antigen levels of value in curative management of colorectal cancer? *Surgery*, **89**, 290.

WEISZ-CARRINGTON, P., POGER, M. E. & LAMM, E. (1976) Secretory immunoglobulins in colonic neoplasms. *Am. J. Pathol.*, **85**, 303.

ZAMCHECK, N., DOOS, W. G., PRUDENTE, R., LURIE, B. B. & GOTTLIEB, L. S. (1975) Prognostic factors in colon carcinoma. Correlation of serum carcinoembryonic antigen level and tumor histopathology. *Human Pathol.* **6**, 31.